# Exposure to Acute Psychological Trauma Prior to Blast Neurotrauma Results in Alternative Behavioral Outcomes

**DOI:** 10.1523/ENEURO.0026-24.2025

**Published:** 2025-03-25

**Authors:** Jessica Strickler, Susan Murphy, Kathryn Athanasaw, Natalia Bowyer, Pamela J. VandeVord

**Affiliations:** ^1^Department of Biomedical Engineering and Mechanics, Virginia Tech, Blacksburg, Virginia 24061; ^2^Veterans Affairs Medical Center, Salem, Virginia 24153; ^3^School of Neuroscience, Virginia Tech, Blacksburg, Virginia 24061; ^4^School of Biomedical Engineering and Sciences, Virginia Tech, Blacksburg, Virginia 24061

**Keywords:** anxiety, blast TBI, depression, stress, TBI

## Abstract

Stress is a common occurrence for military personnel. This can include the stress of deployment and active combat. Anxiety is considered a reaction to stress, and with anxiety-related disorders on the rise, it is imperative that stress be considered a preexisting condition when studying a number of neurological conditions. To determine the effects of stress on the behavioral outcomes of traumatic brain injury (TBI), we used a 3 d acute unpredictable stress (AUS) model followed by blast-induced neurotrauma (BINT) to assess social anhedonia and anxiety-like behaviors in male and female rats. The animals were divided into four groups including unstressed and uninjured control (Con), stress-only animals (AUS), injury-only animals (BINT), and animals that received both stress and injury (AUS + BINT). In the males, behavioral tests such as elevated plus and three-chamber sociability (3-CS) showed that stress plays a dominant role in determining behavioral outcomes after TBI with the AUS + BINT animals behaving more similarly to the AUS animals than the BINT animals. Other tests, such as open field, showed that AUS + BINT had an additive effect on anxiety-like behavior or that prestress could even have a protective effect as seen in three-chamber social novelty (3-CSN). Behavioral assessment of female animals showed that AUS + BINT had the opposite effect than it did on the males in both three-chamber sociability and three-chamber social novelty, while the open field results were similar to the males. This study shows that neurological changes driven by stress have an effect on the behavioral outcomes of BINT.

## Significance Statement

It has been well established that exposure to even acutely stressful situations can cause long-lasting neurological and behavioral changes. While many studies have focused on the neuropathological and psychological aspects of traumatic brain injury (TBI) and stress separately, the relationship between the two is understudied. Current preclinical models of TBI actively attempt to minimize the animal's exposure to stress to prevent any stress-induced neurological changes from interfering with TBI-related outcomes. Here, we demonstrate that, by not factoring in stress-induced neurological changes, we are limiting the clinical relevancy of the TBI model given that stress is an everyday factor in human populations.

## Introduction

Combat and operational stress can include psychological distress linked to mobilization, family separation, and the anticipation of combat leading to cognitive, emotional, behavioral, and physiological responses of service members to stress in combat and military operations other than war (Department of the Army). This suggests that the mental state of these soldiers is already affected before ever experiencing actual combat and has led to a surge in behavioral health issues (e.g., anxiety, irritability, aggression, depression, and substance abuse) throughout the military (Breitbach et al., 2018; Maglione et al., 2021). Traumatic brain injury (TBI) is another significant contributor to these disorders, affecting roughly 2.8 million people in the USA annually, with mild TBI (mTBI) accounting for over 80% of all cases. In the military, TBI is a significant concern, with 375,230 cases diagnosed among US forces from 2,000 through September 2017, including 220,014 cases among the army (Kong et al., 2022). Individuals with military-related TBI are at a higher risk for mental health conditions, including posttraumatic stress disorder, depression, and anxiety (Howard et al., 2022). For instance, 25.6% of soldiers with a TBI had new-onset anxiety disorders, compared with 9.8% of soldiers without a TBI (Brenner et al., 2023). TBI has also been linked to increased risks of substance use disorders, mood disorders, and suicide among military personnel (Brenner et al., 2023).

Previous studies showed that a history of psychiatric disorders before the TBI was common in TBI survivors, with approximately 38% having preinjury Axis I disorders, 19% having a history of anxiety disorders, and 13% having a history of depressive disorders pre-TBI (Scholten et al., 2016). A history of psychiatric disorders pre-TBI was significantly associated with a higher risk of developing or worsening psychiatric disorders post-TBI (Scholten et al., 2016). Despite the prevalence of both stress and TBI and similarities of their outcomes, little is known about how stress and TBI interact.

Few studies have looked at the effects of prestress on TBI outcomes. Some studies have looked at the effects of early life stress (ELS), through maternal separation on TBI. Male Sprague Dawley (SD) rats experienced ELS for 3 h a day, on postnatal days 2–14, and then received mild to moderate fluid percussion injury at 2 months of age (Sanchez et al., 2021; Zheng et al., 2022). Behavioral testing found that the combination of ELS and a later TBI resulted in adulthood impaired hippocampal-dependent learning suggesting ELS is a risk factor that can exacerbate post-TBI outcomes (Sanchez et al., 2021; Zheng et al., 2022). Other studies of ELS prior to TBI revealed decreased spatial learning and memory deficits (Diaz-Chavez et al., 2020; Zheng et al., 2022). Another study that used social defeat stress on adult male Sprague Dawley rats up to 30 min prior to an mTBI found that rats who experienced both social defeat and mTBI exhibited grater impaired contextual fear extinction compared with social defeat stress- or mTBI-only rats (Davies et al., 2016; Zheng et al., 2022). All of these studies were limited to male animals.

In this study, male and female animals were exposed to 3 d of unpredictable stress immediately followed by 3 d of blast-induced neurotrauma (BINT) to investigate the effects of stress-induced neurological changes on long-term behavioral outcomes of a diffuse model of TBI. Social anhedonia (SA) and anxiety-like behaviors have previously been studied in stress-alone or injury-alone models, but this is one of the first studies to assess a prestressed TBI model. To determine the behavioral changes that are associated with stress, TBI, and stress combined with TBI, we used three different behavioral tests including three-chamber sociability, elevated plus maze, and open field. These findings indicated that, in many aspects of behavioral testing, stress plays a significant role in the outcome of TBI behavioral changes. Additionally, in many of the behavioral tests, we found that the females reacted differently to prestress than the males did. Together, this suggests that future TBI-related studies should consider stress as a preexisting condition and further supports the importance of sex-specific studies.

## Materials and Methods

### Animals

**Figure 1.**
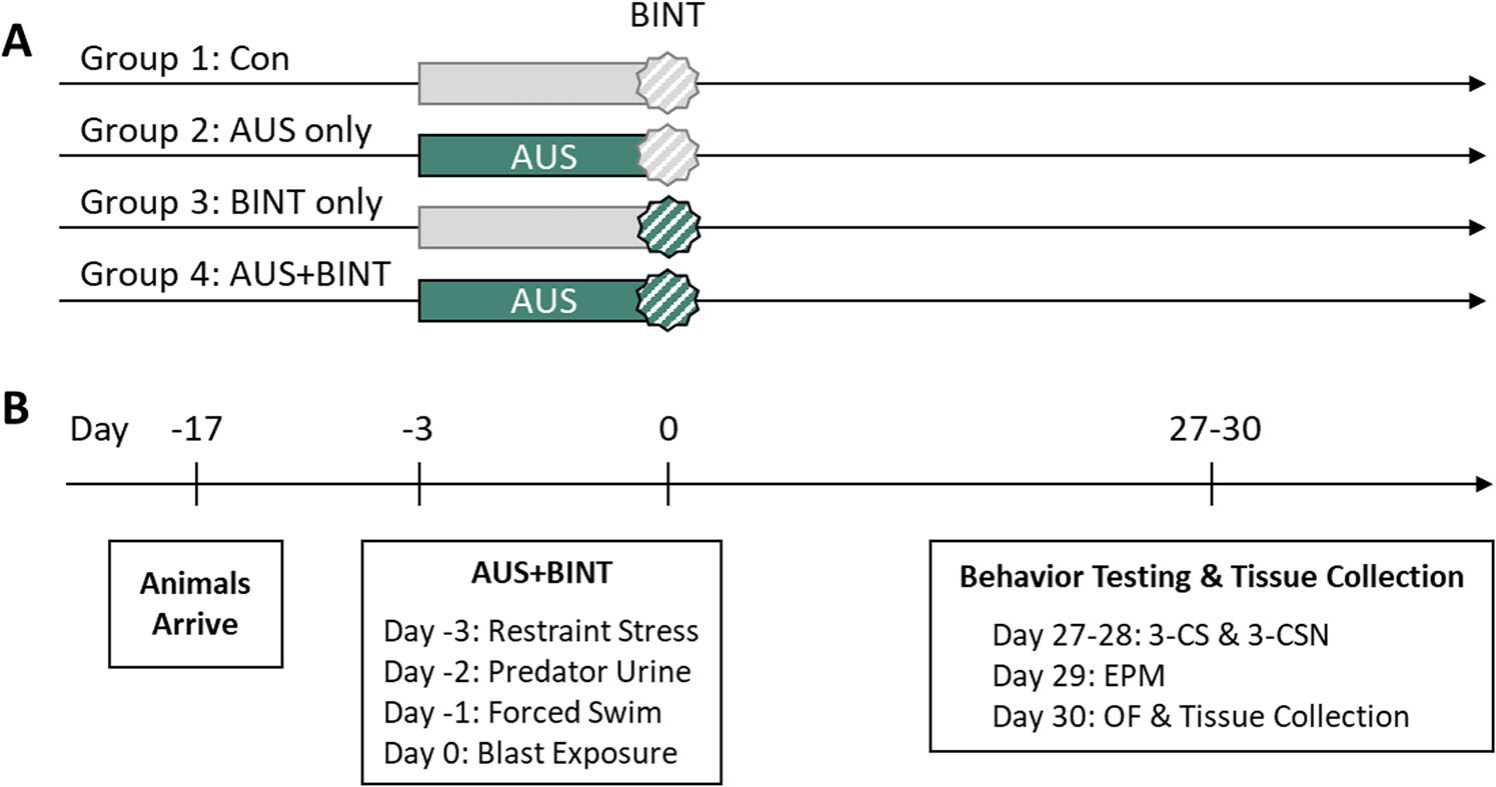
Study design. ***A***, Animals were randomly assigned to one of four groups: Con, AUS only, BINT only, or AUS + BINT. ***B***, Animals arrived and were acclimated for 12–14 d. They were exposed to 3 d of alternating stressors and 3 d of a single blast exposure. The animals were allowed to recover for 27 d before behavior testing.

All procedures were conducted following approval from the Virginia Tech Institutional Animal Care and Use Committee. Adult, male and female Sprague Dawley (SD) rats (*n* = 10–18 animals per group) were acclimated for 2 weeks on a 12 h light/dark cycle. Rats were maintained at 70°F and 70% humidity and had *ad libitum* access to food and water. Male and female rats were run and analyzed separately. All animals were randomly assigned to one of the four treatment groups: (1) control (Con); (2) stress only (AUS); (3) blast-induced neurotrauma (BINT); (4) stress plus blast-induced neurotrauma (AUS + BINT; Fig. 1*A*). During acclimation, all animals were tattooed and weighed. To limit stress unrelated to the experimental paradigm, rats were handled daily to minimize handling-related stress and pair-housed to alleviate complications of isolation stress (Patterson-Kane et al., 2004; Flak et al., 2009; Ferland and Schrader, 2011).

### Acute unpredictable stress (AUS)

Rats belonging to either the AUS or AUS + BINT groups were exposed to a single alternating stressor once a day for 3 d so as not to habituate to any one stressor (Fig. 1*B*). Stress sessions were conducted between 9 A.M. (ZT0) and 11 A.M. (ZT2) each day. Day 1 consisted of a restraint stress; animals were placed in a DecapiCone and secured for 1 h in their home cage. After 1 h, the animals were released into the home cage. Day 2 consisted of predator urine exposure; animals were placed in individual mouse cages where they were exposed to 1 ml of synthetic fox urine pipette onto a nonwoven sponge and taped to the inside wall of the cage with loud noises and sudden cage movements at irregular times as an unpredictable stressor. After 15 min, the rats were returned to their home cage. Day 3 consisted of forced swim stress; animals were placed one at a time in a swim tank containing 30 cm deep, room temperature water and monitored continuously for 10 min. After the 10 min concluded, the animals were dried off and placed in their home cage, on a heating pad for 30 min. Con and BINT animals were lightly handled during each stress day to act as a sham stress.

### Blood collection and corticosterone ELISAs

Blood was collected from six animals from each group at 60–90 min following the conclusion of forced swim stress. Roughly 200 μl of blood was collected with a tail nick procedure using Microvette 200 capillary blood collection tubes (Sarstedt) and centrifuged for 10 min at 1,000–2,000 × *g* using a table top refrigerated centrifuge. The serum was collected and processed for evaluation of corticosterone (Cort) levels using the Neogen corticosterone ELISA kit (Neogen). Results were calculated as recommended by Neogen through MyAssays.com.

### Blast exposure

Blast and sham exposures occurred the day after the final stressing session. Blast and sham sessions were conducted between 9 A.M. (ZT0) and 12 P.M. (ZT3) each day. All animals were segregated according to stress treatment in remote, quiet locations away from the advanced blast simulator (ABS). Prior to testing, each animal was administered general anesthesia (4% isoflurane in the infusion chamber). The ABS (200 cm × 30.48 cm × 30.48 cm) consisted of a driving compression chamber attached to a rectangular transition and testing chamber. The end of the ABS contains a passive end-wave eliminator that minimizes shock wave outflow and rarefaction back through the chamber. The shock wave was generated by the compression of helium (driver) and the rupture of calibrated acetate sheets. Pressure measurements were collected at 500 kHz using a Dash 8HF data acquisition system (Astro-Med). Blast treatment consisted of three anterior blasts [average positive phase of peak static overpressure in males, 136.6 ± 12.5 kPa (19.8 ± 2.0 psi), and females, 124.0 ± 19.6 kPa (17.11 ± 3.0 psi); duration, ∼2 ms] at 24 h intervals with the anesthetized animal suspended in the prone position. Recovery from anesthesia was monitored on a warming pad until the rat was fully mobile. Anesthetized shams underwent the same procedures with the exception of the blast; instead, they endured three aversive auditory stimuli produced in close proximity to the ABS.

### Three-chambered sociability and social novelty (social anhedonia)

Animals performed a three-chambered test at 27–28 d postinjury and was carried out in three sequential trials. Sessions were conducted between 9 A.M. (ZT0) and 5 P.M. (ZT8). The arena for this test has three chambers, with a cylindrical cage in the first and third chambers with the center chamber remaining empty. In the first trial, the rat was given a 5 min habituation period with the doors open and both cages empty. For the sociability test (3-CS), the test rat was immediately placed in the center chamber with the doors in place, and an unfamiliar rat (first stranger) was placed in a cage in the first side chamber with acrylic bars that allow nose contact between the bars. The opposite (third) chamber contained an identical, empty cage. The test rat was allowed to explore the arena freely for 10 min. In the social novelty test (3-CSN), the test rat was placed back into the center chamber with the doors in place, the first stranger (now a familiar rat) was left in its cage, and a novel rat was placed in the cage in the third chamber. The doors were then removed, and the test rat was allowed to explore the entire arena for 10 min. The tests were recorded in standard room lighting, an isolated room with an overhead mounted infrared camera. Video files were captured and saved for later scoring using EthoVision XT (Noldus Information Technology) video tracking software. Scored behaviors include total distance, average velocity, total time spent in each chamber, total time spent in the “sniff zone” designated by a circular area 1.25 times the size of the base of the stranger cages, and preference for each chamber and sniff zone. Preference for chamber and sniff zone was calculated as follows: (total time spent in stranger/novel) ÷ [(total time spent in stranger/novel) + (total time spent in empty/familiar)]. A value of >0.5 represents the preference for the stranger or novel animal while a value of <0.5 represents a preference for the empty or familiar animal.

### Elevated plus maze

Animals performed elevated plus maze (EPM) at 29 d postinjury, sessions were conducted between 9 A.M. (ZT0) and 1 P.M. (ZT4). The rat-configured, plastic EPM apparatus consists of two open arms (50 cm × 10 cm × 50 cm), two enclosed arms (50 cm × 10 cm × 30 cm enclosures), and a center square region (10 cm × 10 cm × 50 cm). The EPM was positioned in an isolated room with standard lighting and an overhead mounted infrared tracking camera to record behavior. Each animal was placed in the center of the maze facing the same open arm (McGuire et al., 2010). Five-minute recordings were initiated as the investigator exits the room (McGuire et al., 2010; Campos et al., 2013). Activity changes were detected using EthoVision XT software. Scored behaviors include total distance moved, average velocity, total time spent in arms (open and enclosed), total number of arm entries (open), total time spent in the center region, and average time per entry (open arm; Pellow and File, 1986; McGuire et al., 2010; Campos et al., 2013).

### Open field (OF)

The animals were acclimated in the open field box before the day before the first AUS stressor. The acclimation ensures that any anxiety-like traits would be due to the exposure to the AUS paradigm or blast and subsequent injury progression. OF assessment was performed at 30 d postinjury, and sessions were conducted between 9 A.M. (ZT0) and 1 P.M. (ZT4). Briefly, animals were placed in an opaque black acrylic box with dimensions 80 cm × 80 cm × 36 cm. Rats were videotaped in an isolated room in standard lighting, using an overhead mounted infrared camera. Activity changes were detected using EthoVision XT software with scored behaviors including total distance moved, average velocity, total time in the center of arena, total number of entries into the center of arena, and average time per entry.

### Statistical analysis

All statistical analyses were performed using Prism 5.0 (GraphPad Software). Serum corticosterone levels were analyzed using a two-way ANOVA to evaluate the main effects of sex and AUS, as well as their interactions. Behavioral test data were analyzed using a three-way ANOVA to evaluate the main effects of sex, AUS, and BINT, as well as their interactions. When the ANOVA indicated significant main effects or interactions, Tukey's post hoc multiple-comparisons test was conducted to identify specific group differences. The sample size (*n*) for each experiment was defined as the number of rats quantified for the respective behavioral test. The exact sample sizes are reported in the figure legends accompanying each experiment. Statistical significance was determined at the *α* = 0.05 level. Results with *p* < 0.05 were considered significant. Statistical significance levels are indicated in the figures as follows: *p < 0.05, **p < 0.01, ***p < 0.001, and ****p < 0.0001. Data are presented as mean ± standard error of the mean (SEM) unless otherwise stated. Table 1 summarizes the statistical analyses used.

**Table 1.** Description of statistical analysis for each figure and corresponding figure panel

Figure	Panel	Data structure	Test used	Degrees of freedom
2	-	Lognormal distribution	Two-way ANOVA	20
3	A	Normal distribution	Three-way ANOVA	86
	B	Normal distribution	Three-way ANOVA	86
	C	Normal distribution	Three-way ANOVA	86
	D	Non-normal distribution	Three-way ANOVA	86
4	A	Normal distribution	Three-way ANOVA	84
	B	Normal distribution	Three-way ANOVA	84
	C	Normal distribution	Three-way ANOVA	84
	D	Normal distribution	Three-way ANOVA	84
5	A	Normal distribution	Three-way ANOVA	88
	B	Normal distribution	Three-way ANOVA	88
	C	Normal distribution	Three-way ANOVA	88
	D	Normal distribution	Three-way ANOVA	88
	E	Normal distribution	Three-way ANOVA	88
	F	Normal distribution	Three-way ANOVA	88
	G	Normal distribution	Three-way ANOVA	88
6	A	Normal distribution	Three-way ANOVA	87
	B	Normal distribution	Three-way ANOVA	87
	C	Normal distribution	Three-way ANOVA	87
	D	Normal distribution	Three-way ANOVA	87
	E	Normal distribution	Three-way ANOVA	87

## Results

### AUS treatment caused increased serum Cort levels in male and female rats

**Figure 2.**
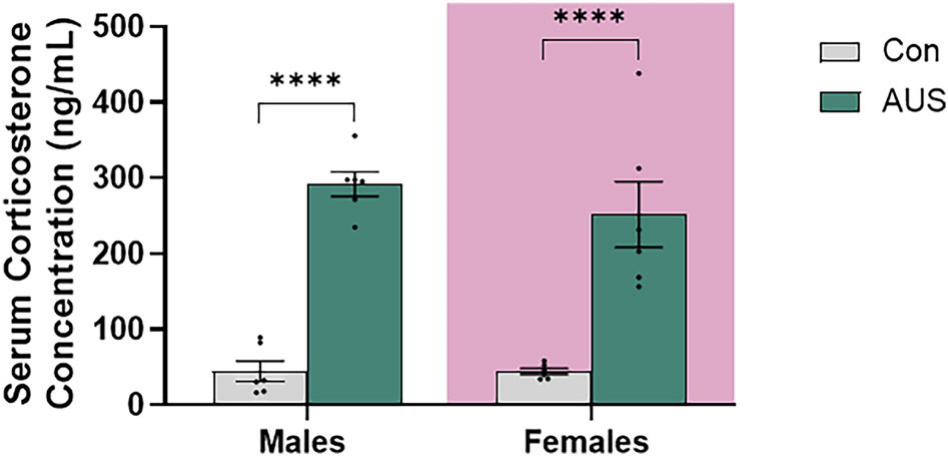
AUS treatment causes increased corticosterone levels. Serum collected 60–90 min following the final AUS treatment revealed increased corticosterone levels in males and females (Con *n* = 6, AUS *n* = 6).

Serum was isolated from blood collected 60–90 min following the completion of the final stressor, forced swim stress, and total serum Cort levels were measured with ELISA analysis. Two-way ANOVA analysis of Cort levels revealed a significant main effect of AUS (Fig. 2; *p* < 0.0001, *F*_(1,20)_ = 87.47), indicating that AUS substantially influenced Cort levels in both males and females. The absence of a significant main effect of sex (*p* = 0.4121, *F*_(1,20)_ = 0.7019) or significant interaction between sex and AUS (*p* = 0.4222, *F*_(1,20)_ = 0.6714) reveals the limited effect of sex on Cort levels. Post hoc analysis of serum corticosterone levels revealed that AUS is sufficient to cause increased Cort levels in both male and female animals (*p* < 0.0001 and *p* = 0.0008, respectively); additionally, there were no sex differences in either of the Con (*p* = 0.9897) or AUS (*p* = 0.2551) group.

### BINT males were more socially motivated than AUS and AUS + BINT animals while BINT females show decreased social preference than Con animals during 3-CS

**Figure 3.**
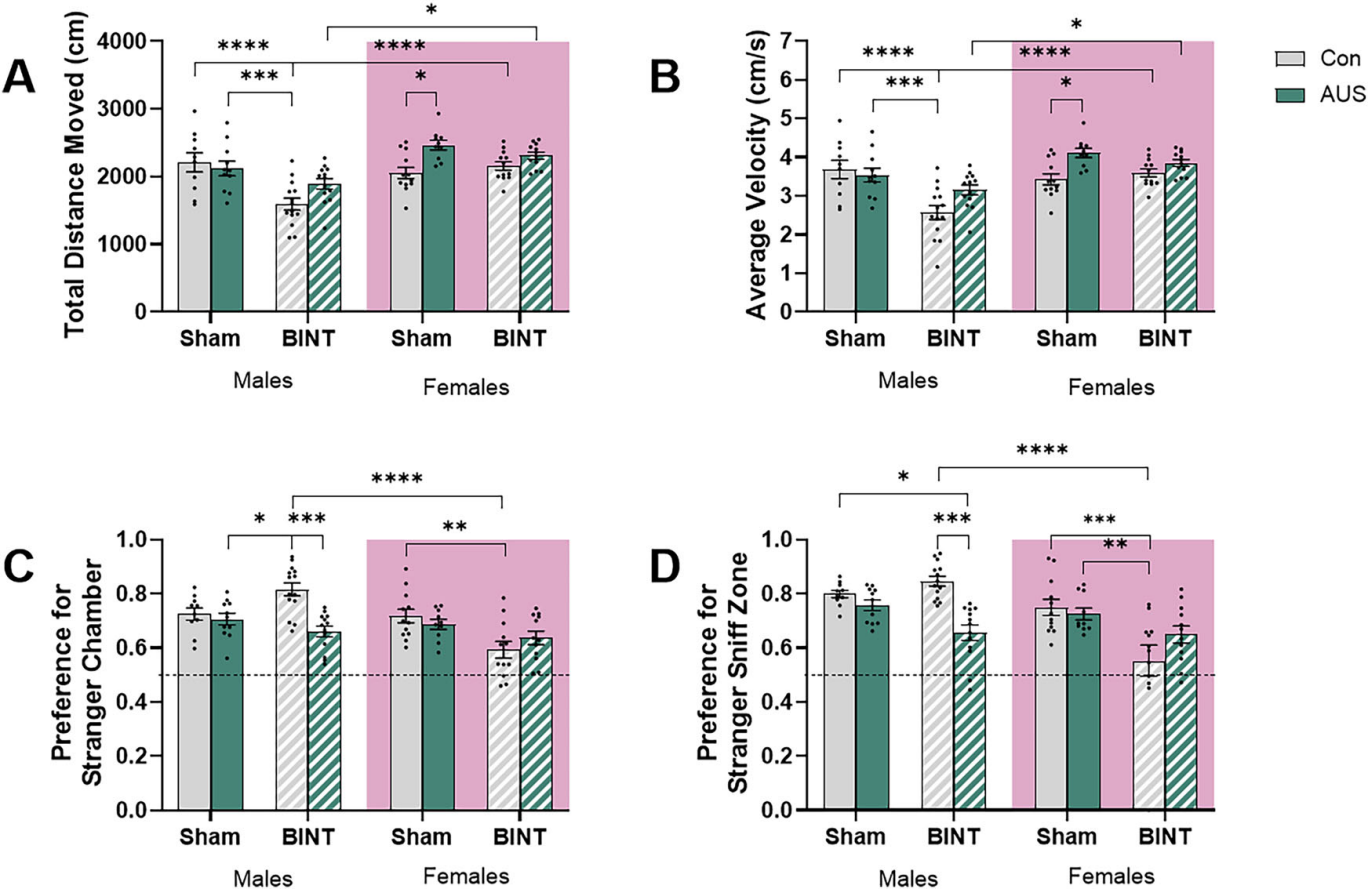
3-CS testing of male and female Sprague Dawley rats 27–28 d postinjury revealed BINT plays a role in social motivation and sex differences. ***A***, Total distance moved measured in centimeters. ***B***, Average velocity measured in centimeters per second. ***C***, Preference for stranger's chamber over the empty chamber. ***D***, Preference for the stranger's sniff zone compared with the empty cage's sniff zone (males, Con *n* = 10, AUS *n* = 11, BINT *n* = 14, AUS + BINT *n* = 13; females, Con *n* = 12, AUS *n* = 10, BINT *n* = 12, AUS + BINT *n* = 12).

Analysis of total distance moved during 3-CS in males and females resulted in major effects of AUS, BINT, and sex (Fig. 3*A*). The three-way ANOVA analysis of total distance moved revealed significant main effects of BINT (*p* = 0.0005, *F*_(1,86)_ = 12.96), AUS (*p* = 0.0021; *F*_(1,86)_ = 9.509), and sex (*p* < 0.0001; *F*_(1,86)_ = 21.89) indicating that all three substantially influenced the animals’ movement patterns individually. There was a significant interaction between BINT and sex (*p* = 0.0021, *F*_(1,86)_ = 10.03) but none between AUS and BINT (*p* = 0.6089, *F*_(1,86)_ = 0.2637), or AUS and sex (*p* = 0.1585; *F*_(1,86)_ = 2.024) suggesting that BINT outcomes are likely affected by sex whereas the effects of AUS is not significantly affected by BINT or sex. Interestingly, there is a significant three-way interaction between sex, AUS, and BINT (*p* = 0.0113, *F*_(1,86)_ = 6.710) observed, implying that the combined effects of AUS and BINT on locomotion differ between males and females. This complex interaction underscores the importance of considering sex as a biological variable in neurotrauma and AUS research. Post hoc comparisons further elucidated these findings. In male animals, there was a significant difference between Con and BINT animals (*p* < 0.0001) and a significant reduction in distance moved between BINT and AUS animals (*p* = 0.001). There were no significant differences between Con and AUS + BINT (*p* = 0.2014), Con and AUS (*p* = 0.9972), BINT and AUS + BINT (*p* = 0.1823), or AUS and AUS + BINT (*p* = 0.5844). In female animals, a different pattern emerged. There were significant differences between Con and AUS groups (*p* = 0.0397), with AUS females moving further. There were no significant differences between Con and BINT (*p* = 0.9905), Con and AUS + BINT (*p* = 0.4415), BINT and AUS + BINT (*p* = 0.919), AUS and BINT (*p* = 0.25770, or AUS and AUS + BINT (*p* = 0.925) females. Analysis of sex differences revealed significant differences between BINT males and females (*p* = 0.0002) and AUS + BINT males and females (*p* = 0.0182), with the females on average moving further. There were no significant differences between Con (*p* = 0.9261) or AUS (*p* = 0.1616) males and females. This pattern suggests that while male and female animals may exhibit similar baseline locomotor activity, their responses to AUS and the combination of both AUS and BINT are markedly different.

Similar to distance, analysis of average velocity during 3-CS in males and females resulted in major effects of AUS, BINT, and sex (Fig. 3*B*). The three-way ANOVA analysis of average velocity revealed a significant main effect of BINT (*p* = 0.0005, *F*_(1,86)_ = 13.25), AUS (*p* = 0.0023; *F*_(1,86)_ = 9.906), and sex (*p* < 0.0001, *F*_(1,86)_ = 21.83) suggesting that all three factors substantially influenced the animals’ movement speed individually. There was a significant interaction between BINT and sex (*p* = 0.0018, *F*_(1,86)_ = 10.42) but none between AUS and BINT (*p* = 0.4915, *F*_(1,86)_ = 0.4772) or AUS and sex (*p* = 0.2494; *F*_(1,86)_ = 1.345) suggesting that BINT outcomes were likely affected by sex whereas the effects of AUS were not significantly affected by BINT exposure or sex. Interestingly, there was a significant three-way interaction between sex, AUS, and BINT observed (*p* = 0.0089, *F*_(1,86)_ = 7.160), implying that the combined effects of AUS and BINT on locomotion differ between males and females. Post hoc comparisons further elucidated these findings. In males, there were significant differences between Con and BINT (*p* < 0.0001) and AUS and BINT (*p* = 0.0004) animals with the BINT males moving significantly slower. There were no significant differences between Con and AUS (*p* = 0.9979), Con and AUS + BINT (*p* = 0.2508), BINT and AUS + BINT (*p* = 0.0884), or AUS and AUS + BINT (*p* = 0.6392) males. In female animals, a different pattern emerged. There were significant differences between Con and AUS groups (*p* = 0.0481), with AUS females moving faster. There were no significant differences between Con and BINT (*p* = 0.9927), Con and AUS + BINT (*p* = 0.5013), BINT and AUS + BINT (*p* = 0.9353), AUS and BINT (*p* = 0.3122), or AUS and AUS + BINT (*p* = 0.9403) females. Analysis of sex differences revealed significant differences between BINT (*p* < 0.0001) and AUS + BINT (*p* = 0.0285) males and females, coupled with the lack of significant differences in the Con (*p* = 0.9411) and AUS (*p* = 0.2061) groups, suggesting that the impact of neurotrauma on average velocity was sex-dependent. This indicated differential vulnerability or coping mechanisms between males and females in response to BINT.

To assess social behavior, analysis of chamber preference during 3-CS resulted in major effects of sex and AUS on male and female animals (Fig. 3*C*). The three-way ANOVA revealed significant main effects of AUS (*p* = 0.0199, *F*_(1,86)_ = 5.629) and sex (*p* = 0.0001, *F*_(1,86)_ = 16.09) suggesting that AUS and sex alone had a substantial impact on the animals’ social behavior. Interestingly, while BINT alone did not show a significant main effect (*p* = 0.0624, *F*_(1,86)_ = 3.563), there was a significant interaction between sex and BINT (*p* = 0018, *F*_(1,86)_ = 10.37) suggesting the effect of BINT was dependent on sex. There was also a significant effect of the interaction between sex and AUS (*p* = 0.007, *F*_(1,86)_ = 7.621) and sex, AUS, and BINT (*p* = 0.0027, *F*_(1,86)_ = 9.537). There was no significant effect of the interaction between BINT and AUS (*p* = 0.3561, *F*_(1,86)_ = 0.8608). The post hoc analysis further elucidated these relationships. In males, BINT showed a significantly higher preference for the stranger's chamber than the AUS (*p* = 0.0268) and AUS + BINT animals (*p* = 0.0001), suggesting that the AUS exposure caused increased social anxiety and highlighting that the behavioral effects of BINT were distinctly different from those of AUS alone or the combination of AUS and BINT. The lack of significant differences between the Con and AUS (*p* = 0.9995), Con and BINT (*p* = 0.1425), Con and AUS + BINT (*p* = 0.5715), and AUS and AUS + BINT groups (*p* = 0.8729) supports that AUS, either alone or in combination with BINT, did not significantly alter behavior compared with Con conditions in male animals. In female animals, the results depicted an opposing response. Only the BINT females showed a significantly lower preference for the stranger chamber compared with the Con females (*p* = 0.0088), further supporting the impact of BINT on female behavior. The lack of significant differences between Con and AUS (*p* = 0.9888), Con and AUS + BINT (*p* = 0.2548), BINT and AUS (*p* = 0.148), BINT and AUS + BINT (*p* = 0.8981), AUS and AUS + BINT (*p* = 0.8412) supports that AUS, either alone or in combination with BINT, did not significantly alter behavior compared with Con or BINT conditions in female animals. Analysis of sex differences within groups revealed a significant difference between BINT males and females only (*p* < 0.0001). This striking result suggests that BINT had markedly different effects on behavior in males and females, while Con (*p* > 0.9999), AUS alone (*p* = 0.9995), or AUS + BINT (*p* = 0.9963) did not produce significant sex differences. This is in part due to the opposing effects of BINT on chamber preference between males and females.

To further assess sociability, assessment of sniff zone preference during 3-CS resulted in major effects of sex and BINT on male and female animals (Fig. 3*D*). The three-way ANOVA revealed a strong main effect of BINT (*p* = 0.0004, *F*_(1,86)_ = 13.64) and sex (*p* < 0.0001, *F*_(1,86)_ = 18.56) on sniff zone preference. This suggested that BINT and sex significantly altered the animals’ behavior in the sniff zone. Interestingly, AUS alone did not show a significant effect (*p* = 0.0757, *F*_(1,86)_ = 3.233), indicating that AUS by itself did not substantially influence sniff zone preference. There were significant interactions between sex and BINT (*p* = 0.016, *F*_(1,86)_ = 6.043) and sex and AUS (*p* = 0.0009, *F*_(1,86)_ = 11.84) suggesting sex has a significant impact on response to BINT and AUS. The interaction between AUS and BINT was not significant (*p* = 0.7604, *F*_(1,86)_ = 0.09360), suggesting that the effect of AUS on sniff zone preference was not significantly modulated by the presence of BINT, and vice versa. There was also a significant effect of the three-way interaction between sex, BINT, and AUS (*p* = 0.0031, *F*_(1,86)_ = 9.248) suggesting a complex interplay between all three factors The post hoc analysis provided further insights. AUS + BINT males showed a significant decrease in sniff zone preference for the stranger compared with their Con (*p* = 0.0394) and BINT (*p* = 0.0003) counterparts. There was a lack of significant differences between Con and BINT (*p* = 0.9634), further supporting the minimal impact of BINT alone on this behavior in males. There were also no significant differences between Con and AUS (*p* = 0.9854), AUS and BINT (*p* = 0.4469), or AUS and AUS + BINT (*p* = 0.2945). In contrast to males, BINT females had a significant reduction in preference for the stranger's sniff zone compared with Con females (*p* = 0.0005), supporting the impact of BINT on female behavior. There was also a significant difference between AUS and BINT females (*p* = 0.0068) with BINT females showing less preference for the stranger than AUS females. The lack of significant differences between Con and AUS (*p* = 0.9995), Con and AUS + BINT (*p* = 0.3103), BINT and AUS + BINT (*p* = 0.347), or AUS and AUS + BINT (*p* = 0.7151) reinforced the minimal impact of AUS on this behavior in females. Assessment of sex differences revealed a significant difference between males and females only in the BINT group (*p* < 0.0001) with the males showing increased preference and the females showing decreased interaction with the stranger. This striking result suggested that BINT had markedly different effects on sniff zone preference in males and females, while Con (*p* = 0.9571), AUS (*p* = 0.9971), or AUS + BINT (*p* > 0.9999) did not produce significant sex differences.

### Male BINT animals do not seek social novelty during 3-CSN while BINT females sought out social novelty at a higher rate than AUS + BINT females

**Figure 4.**
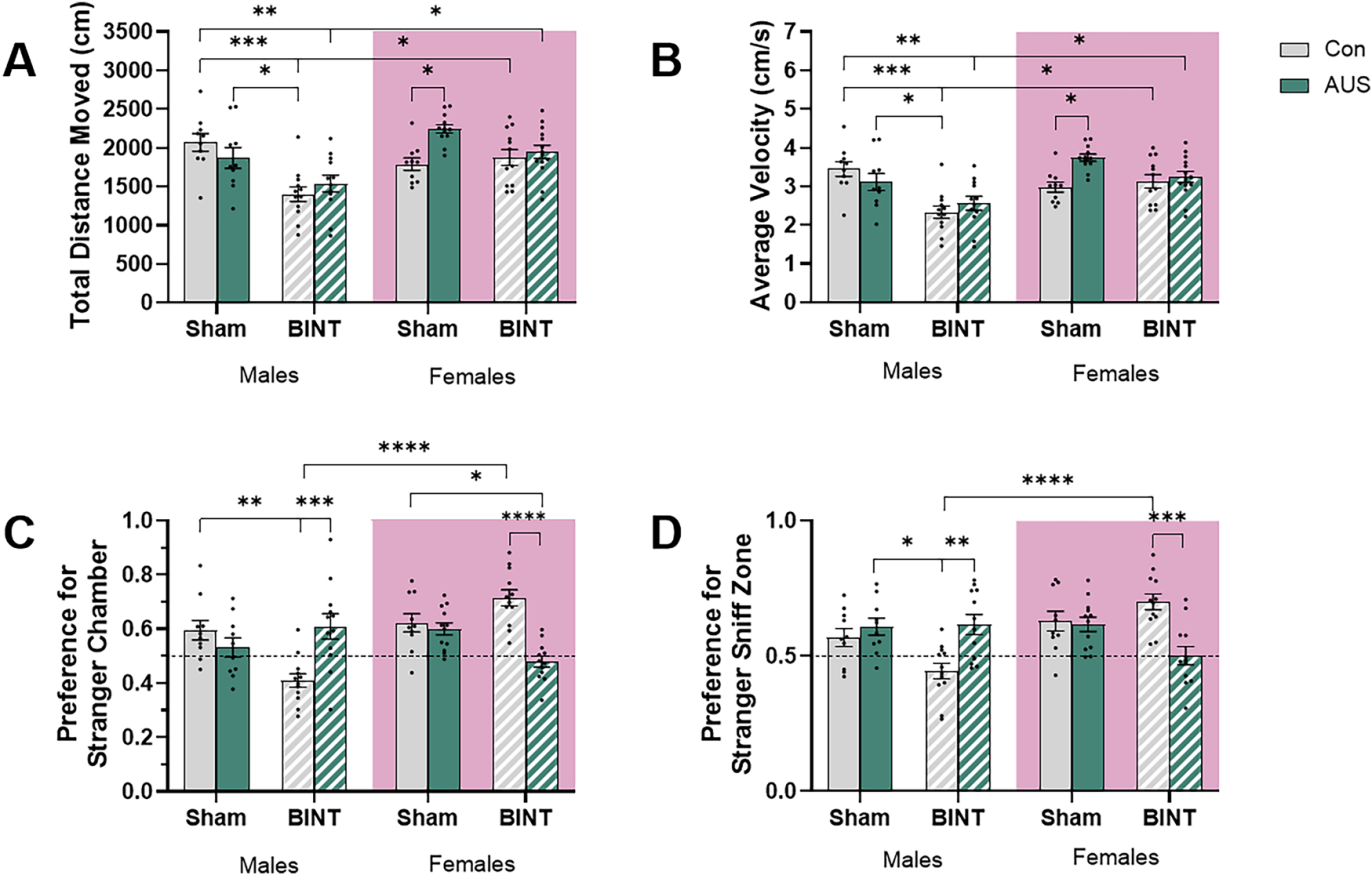
3-CSN testing of male and female Sprague Dawley rats 27–28 d postinjury revealed BINT and AUS + BINT driven sex differences in preference for the novel animal compared with other groups. ***A***, Total distance moved measured in centimeters. ***B***, Average velocity measured in centimeters per second. ***C***, Preference for novel animal's chamber over the familiar animal's chamber. ***D***, Preference for the novel animal's sniff zone compared with the familiar animal's sniff zone (males, Con *n* = 10, AUS *n* = 10, BINT *n* = 12, AUS + BINT *n* = 12; females, Con *n* = 10, AUS *n* = 12, BINT *n* = 12, AUS + BINT *n* = 14).

Analysis of average velocity during 3-CSN in males and females resulted in major effects of BINT and sex (Fig. 4*A*). The three-way ANOVA revealed a significant main effect of BINT (*p* < 0.0001, *F*_(1,84)_ = 18.91) and sex (*p* = 0.0007, *F*_(1,84)_ = 12.39) on total distance moved. However, there was no main effect of AUS (*p* = 0.1016, *F*_(1,84)_ = 2.740) as well as no interaction between AUS and BINT (*p* = 0.8586, *F*_(1,84)_ = 0.03193). However, there was a significant two-way interaction between sex and BINT (*p* = 0.0057, *F*_(1,84)_ = 8.044) and sex and AUS (*p* = 0.0373, *F*_(1,84)_ = 4.480) suggesting sex has an effect on both BINT and AUS individually. Additionally, there was a three-way interaction between sex, AUS, and BINT (*p* = 0.0106, *F*_(1,84)_ = 6.831) implying that the combined effects of AUS and BINT on locomotion differ between males and females. Post hoc multiple comparisons for male animals showed that compared with Con males, BINT (*p* = 0.0002), and AUS + BINT (*p* = 0.0075) males significantly reduced the total distance moved. BINT males also moved a shorter distance than AUS males (*p* = 0.0298) while there was no significant difference between AUS and AUS + BINT males (*p* = 0.291) or BINT and AUS + BINT males (*p* = 0.971) with the AUS + BINT males moving with a distance shorter than the AUS animals but longer than the BINT animals but not significantly so. We also observed nonsignificant differences between Con and AUS (*p* = 0.8728). These nonsignificant results suggested that AUS alone did not substantially alter movement patterns and that the effects of BINT were not significantly modulated by the addition of AUS. For female animals, AUS significantly increased the total distance moved compared with Con females (*p* = 0.0372). However, several comparisons yielded nonsignificant results: Con versus BINT (*p* = 0.9984), Con versus AUS + BINT (*p* = 0.9446), AUS versus BINT (*p* = 0.132), BINT versus AUS + BINT (*p* = 0.9995), and AUS versus AUS + BINT (*p* = 0.3079). These nonsignificant results support the idea that in females, BINT alone did not significantly alter movement and that the combination of AUS and BINT did not produce effects significantly different from Con conditions or BINT alone. Assessment of sex differences revealed significant differences between males and females in the BINT (*p* = 0.0152) and AUS + BINT (*p* = 0.0477) groups with females moving, on average, further than males. This is likely due to the decreased mobility seen in BINT and AUS + BINT males. Notably, no significant sex differences were observed in the Con (*p* = 0.5557) or AUS (*p* = 0.11518) groups, indicating that baseline movement and response to AUS alone were similar between sexes.

Similar to distance, analysis of average velocity during 3-CSN in males and females resulted in major effects of BINT and sex (Fig. 4*B*). The three-way ANOVA revealed a significant main effect of BINT (*p* < 0.0001, *F*_(1,84)_ = 18.83) and sex (*p* = 0.0007, *F*_(1,84)_ = 12.41) on average velocity. This strong effect indicated that BINT substantially altered the animals’ movement speed. However, there was no main effect of AUS (*p* = 0.1031, *F*_(1,84)_ = 2.716). There were significant interactions between sex and AUS (*p* = 0.0379, *F*_(1,84)_ = 4.447) and sex and BINT (*p* = 0.0057, *F*_(1,84)_ = 8.064) suggesting sex plays a role in the reaction to AUS and BINT. While there were no significant interactions between AUS and BINT (*p* = 0.8541, *F*_(1,84)_ = 0.03401), there was a three-way interaction between sex, AUS, and BINT (*p* = 0.0105, *F*_(1,84)_ = 6.850). Post hoc multiple comparisons revealed that for male animals, the BINT (*p* = 0.0003) and AUS + BINT (*p* = 0.0076) males moved significantly slower than Con males. BINT males also moved significantly slower than AUS males (*p* = 0.0301). Importantly, several comparisons yielded nonsignificant results: Con versus AUS (*p* = 0.8733), BINT versus AUS + BINT (*p* = 0.9711), and AUS versus AUS + BINT (*p* = 0.2921). These nonsignificant findings indicated that AUS alone did not substantially alter velocity patterns and that the effects of BINT were not significantly modulated by the addition of AUS. Assessment of female animals showed that AUS significantly increased average velocity compared with Con (*p* = 0.0374). However, several comparisons yielded nonsignificant results: Con versus BINT (*p* = 0.9982), Con versus AUS + BINT (*p* = 0.9448), AUS versus BINT (*p* = 0.1364), BINT versus AUS + BINT (*p* = 0.9996), and AUS versus AUS + BINT (*p* = 0.3089). These nonsignificant results further supported that BINT alone did not significantly alter velocity in females and that the combination of AUS and BINT did not produce effects significantly different from Con conditions or BINT alone. Assessment of sex differences revealed significant differences between males and females in the BINT (*p* = 0.0148) and AUS + BINT (*p* = 0.0481) groups with females traveling faster than their male counterparts. Notably, no significant sex differences were observed in the Con (*p* = 0.5568) or AUS (*p* = 0.1526) groups, indicating that baseline velocity and response to AUS alone were similar between sexes.

To assess social novelty behavior, analysis of chamber preference during 3-CSN revealed that sex, AUS, and BINT had an effect on males and females (Fig. 4*C*). The three-way ANOVA revealed a significant main effect of sex (*p* = 0.0036, *F*_(1,82)_ = 8.999) but no significant main effects of AUS (*p* = 0.1838, *F*_(1,82)_ = 1.797) or BINT (*p* = 0.1339, *F*_(1,82)_ = 2.292). There was also no significant interaction between sex and BINT (*p* = 0.3872, *F*_(1,82)_ = 0.7558) or AUS and BINT (*p* = 0.58, *F*_(1,82)_ = 0.3087). However, there was a significant two-way interaction of sex and AUS (*p* < 0.0001, *F*_(1,82)_ = 19.11) and a significant three-way interaction between sex, AUS, and BINT (*p* < 0.0001, *F*_(1,82)_ = 27.82). Post hoc multiple comparisons further elucidated these findings. For male animals, BINT males showed a significantly decreased preference for the novel animal's chamber compared with Con (*p* = 0.0027) and AUS + BINT (*p* = 0.0004) males, but not AUS males (*p* = 0.1448). These results suggested that BINT alone and the combination of AUS and BINT had distinct effects on chamber preference. However, several comparisons yielded nonsignificant results: Con versus AUS (*p* = 0.887), Con versus AUS + BINT (*p* > 0.9999), and AUS versus AUS + BINT (*p* = 0.685). These nonsignificant findings indicated that AUS alone did not significantly alter chamber preference compared with Con and that the effects of BINT were not significantly different from AUS alone in males. For female animals, AUS + BINT showed a significantly reduced preference for the novel animal's chamber compared with Con (*p* = 0.0459) and BINT (*p* < 0.0001). However, several comparisons yielded nonsignificant results: Con versus BINT (*p* = 0.4793), Con versus AUS (*p* = 0.9997), BINT versus AUS (*p* = 0.1613), and AUS versus AUS + BINT (*p* = 0.1143). These nonsignificant findings suggested that neither AUS nor BINT alone significantly altered chamber preference compared with Con in females. Assessment of sex differences revealed a significant difference between males and females in the BINT group (*p* < 0.0001) where the males showed preference for the familiar animal and the females showed preference for the novel animal. This suggests that BINT affected chamber preference differently in males and females. Notably, no significant sex differences were observed in the Con (*p* = 0.999), AUS (*p* = 0.8058), or AUS + BINT (*p* = 0.0677) groups, indicating that baseline chamber preference, response to AUS alone, and response to the combination of AUS and BINT were similar between sexes.

To further assess social novelty behavior, analysis of sniff zone preference during 3-CSN revealed that sex, AUS, and BINT had an effect on males and females (Fig. 4*D*). The three-way ANOVA revealed a significant main effect of sex (*p* = 0.023, *F*_(1,82)_ = 5.365) but not AUS (*p* = 0.9848, *F*_(1,82)_ = 0.0003655) or BINT (*p* = 0.818, *F*_(1,82)_ = 3.105). There was no two-way interaction between AUS and BINT (*p* = 0.5496, *F*_(1,82)_ = 0.3610) or sex and BINT (*p* = 0.4355, *F*_(1,82)_ = 0.6140). However, there was a two-way interaction between sex and AUS (*p* < 0.0001, *F*_(1,82)_ = 21.48) and a three-way interaction between sex, AUS, and BINT (*p* = 0.0008, *F*_(1,82)_ = 12.14). Post hoc multiple comparisons further elucidated these effects. Analysis of the male animals showed significant differences between AUS and BINT groups (*p* = 0.0138) and between BINT and AUS + BINT groups (*p* = 0.0045) where the BINT males show an increased preference for the familiar animal. These results suggested that BINT alone had distinct effects on sniff zone preference compared with AUS alone or the combination of AUS and BINT. However, several comparisons yielded nonsignificant results: Con versus BINT (*p* = 0.1426), Con versus AUS (*p* = 0.9903), Con versus AUS + BINT (*p* = 0.9964), and AUS versus AUS + BINT (*p* > 0.9999). These nonsignificant findings indicated that neither AUS alone nor the combination of AUS and BINT significantly altered sniff zone preference compared with Con conditions in males. Assessment of the female animals revealed a significant difference between the BINT and AUS + BINT groups (*p* = 0.0005) where BINT females have a higher preference for the novel animal, indicating that the combination of AUS and BINT had a distinct effect compared with BINT alone. However, several comparisons yielded nonsignificant results: Con versus BINT (*p* = 0.7861), Con versus AUS (*p* > 0.9999), Con versus AUS + BINT (*p* = 0.1184), BINT versus AUS (*p* = 0.5646), and AUS versus AUS + BINT (*p* = 0.1621). These nonsignificant findings suggested that neither AUS nor BINT alone significantly altered sniff zone preference compared with Con in females and that the effects of AUS alone were not significantly different from the combination of AUS and BINT. Assessment of sex differences revealed a significant difference between males and females in the BINT group (*p* < 0.0001) where males showed a preference for the familiar animal and females showed a preference for the novel animal, suggesting that BINT affected sniff zone preference differently in males and females. Notably, no significant sex differences were observed in the Con (*p* = 0.9072), AUS (*p* > 0.9999), or AUS + BINT (*p* = 0.1703) groups, indicating that baseline sniff zone preference, response to AUS alone, and response to the combination of AUS and BINT were similar between sexes.

### AUS and AUS + BINT males and AUS females have increased exploratory behavior during EPM

**Figure 5.**
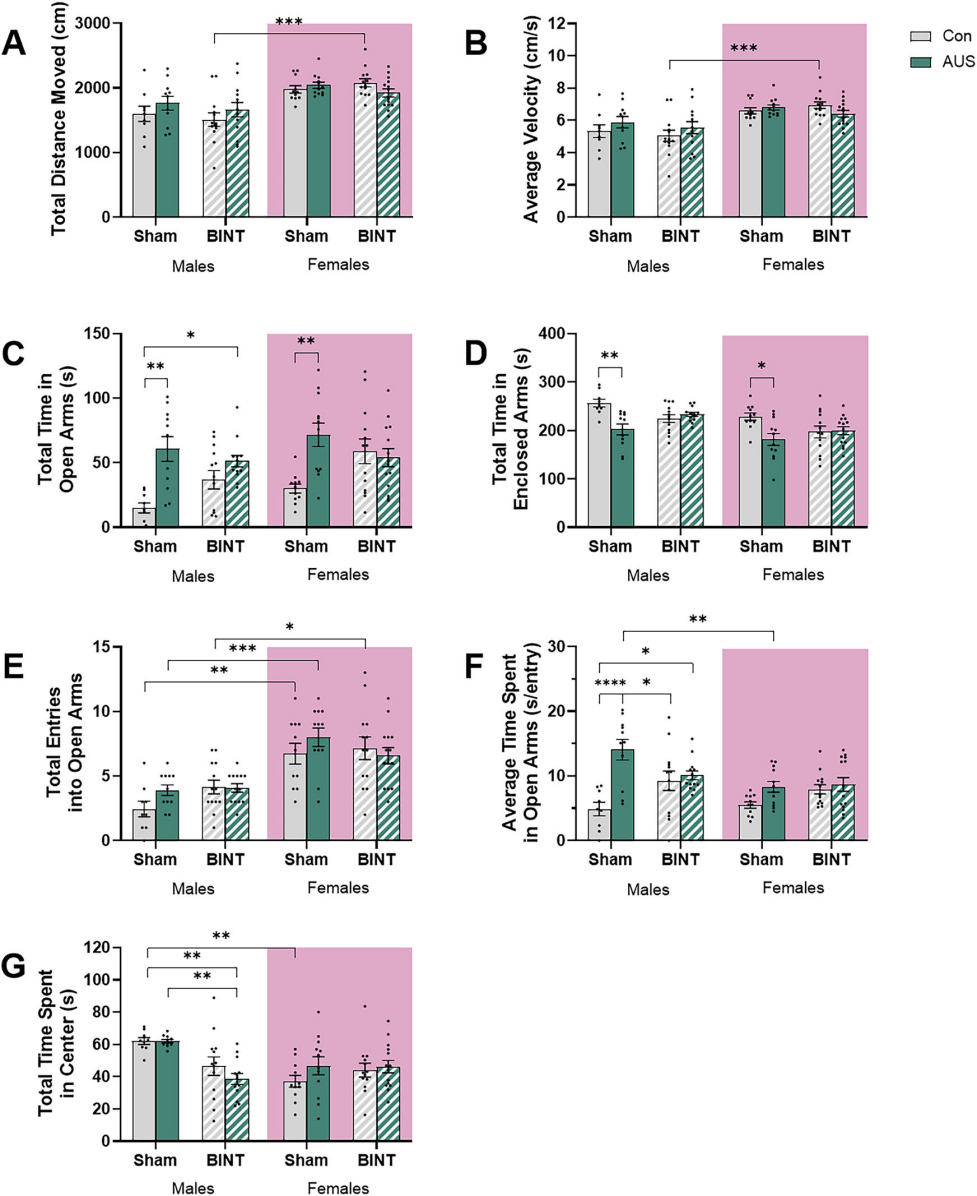
EPM testing of male and female Sprague Dawley rats 29 d postinjury revealed females had more exploratory behavior than their male counterparts. ***A***, Total distance moved measured in centimeters. ***B***, Average velocity measured in centimeters per second. ***C***, Total time spent in the enclosed arms. ***D***, Total time spent in open arms. ***E***, Total number of entries into the open arms. ***F***, Average time per entry into the open arms measured as seconds per entry. ***G***, Total time spent in the center zone of the maze (males, Con *n* = 10, AUS *n* = 11, BINT *n* = 13, AUS + BINT *n* = 13; females, Con *n* = 11, AUS *n* = 12, BINT *n* = 13, AUS + BINT *n* = 14).

Assessment of total distance moved indicated that there were no significant differences between groups within each sex (Fig. 5*A*). A three-way ANOVA revealed a significant main effect of sex (*p* < 0.0001, *F*_(1,88)_ = 36.09) but no main effect of AUS (*p* = 0.3686, *F*_(1,88)_ = 0.8169) or BINT (*p* = 0.4060, *F*_(1,88)_ = 0.6971). Additionally, there were no significant two-way interactions between AUS and BINT (*p* = 0.347, *F*_(1,88)_ = 0.8939), sex and AUS (*p* = 0.1005, *F*_(1,88)_ = 2.754), or sex and BINT (*p* = 0.4811, *F*_(1,88)_ = 0.5006), nor was there a significant three-way interaction between sex, AUS, and BINT (*p* = 0.3989, *F*_(1,88)_ = 0.7185). These nonsignificant interactions suggested that regardless of sex, AUS and BINT have no effect on motor activity during EPM testing. Post hoc multiple comparisons further supported these findings, showing no significant differences between any of the male groups: Con versus BINT (*p* = 0.9972), Con versus AUS (*p* = 0.9241), Con versus AUS + BINT (*p* = 0.9997), BINT versus AUS (*p* = 0.4462), BINT versus AUS + BINT (*p* = 0.8986), and AUS versus AUS + BINT (*p* = 0.9913). Additionally, there were no significant differences between groups in females: Con versus BINT (*p* = 0.9904), Con versus AUS (*p* = 0.9996), Con versus AUS + BINT (*p* = 0.9998), BINT versus AUS (*p* > 0.9999), BINT versus AUS + BINT (*p* = 0.8714), and AUS versus AUS + BINT (*p* = 0.9725). These consistently nonsignificant results suggest that in male and female animals, neither AUS nor BINT, alone or in combination, significantly affected the total distance moved. However, assessment of sex differences revealed significant differences between males and females in the BINT (*p* = 0.0001) with the females traveling further. Interestingly, no significant sex differences were observed in the Con (*p* = 0.1082), AUS (*p* = 0.3536), or AUS + BINT (*p* = 0.3332) groups. These results suggested that while there were inherent sex differences in total distance moved, particularly under BINT conditions, these differences were mitigated in the presence of AUS.

For assessment of average velocity, there were no significant differences between experimental groups within each sex (Fig. 5*B*). A three-way ANOVA revealed a significant main effect of sex (*p* < 0.0001, *F*_(1,88)_ = 36.09) but no main effect of AUS (*p* = 0.3693, *F*_(1,88)_ = 0.8142) or BINT (*p* = 0.408, *F*_(1,88)_ = 0.6912). Additionally, there were no significant two-way interactions between AUS and BINT (*p* = 0.3462, *F*_(1,88)_ = 0.8968), sex and AUS (*p* = 0.1009, *F*_(1,88)_ = 2.748), or sex and BINT (*p* = 0.4833, *F*_(1,88)_ = 0.4955), nor was there a significant three-way interaction between sex, AUS, and BINT (*p* = 0.400, *F*_(1,88)_ = 0.7151). These nonsignificant interactions suggested that regardless of sex, AUS and BINT have no effect on average velocity during EPM testing. Post hoc multiple comparisons further supported these findings, showing no significant differences between any of the male groups: Con versus BINT (*p* = 0.9973), Con versus AUS (*p* = 0.9241), Con versus AUS + BINT (*p* = 0.9997), BINT versus AUS (*p* = 0.4496), BINT versus AUS + BINT (*p* = 0.8999), and AUS versus AUS + BINT (*p* = 0.9914). Additionally, there were no significant differences between groups in females: Con versus BINT (*p* = 0.9904), Con versus AUS (*p* = 0.9996), Con versus AUS + BINT (*p* = 0.9998), BINT versus AUS (*p* > 0.9999), BINT versus AUS + BINT (*p* = 0.8716), and AUS versus AUS + BINT (*p* = 0.9725). These consistently nonsignificant results suggested that in male and female animals, neither AUS nor BINT, alone or in combination, significantly affected the total distance moved. However, assessment of sex differences revealed significant differences between males and females in the BINT (*p* = 0.0001) groups with the females traveling further. Interestingly, no significant sex differences were observed in the Con (*p* = 0.1083), AUS (*p* = 0.3541), or AUS + BINT (*p* = 0.3346) groups. These results suggested that while there are inherent sex differences in total distance moved, particularly under BINT conditions, these differences were mitigated in the presence of AUS.

Assessment of total time spent in the open arms revealed that AUS males and females as well as AUS + BINT males spent more time in the open arms compared with their control counterparts (Fig. 5*C*). A three-way ANOVA revealed significant main effects of AUS (*p* < 0.0001, *F*_(1,88)_ = 21.04) and sex (*p* = 0.0186, *F*_(1,88)_ = 5.750) with no main effect of BINT (*p* = 0.2698, *F*_(1,88)_ = 1.233). Interestingly, there was a two-way interaction between AUS and BINT (*p* = 0.0004, *F*_(1,88)_ = 13.54) but not sex and AUS (*p* = 0.2725, *F*_(1,88)_ = 1.219) or sex and BINT (*p* = 0.9613, *F*_(1,88)_ = 0.002363). Additionally, there was no three-way interaction between sex, AUS, and BINT (*p* = 0.4748, *F*_(1,88)_ = 0.5151). Post hoc multiple comparisons further elucidated these effects. In males, AUS (*p* = 0.0036) and AUS + BINT (*p* = 0.0332) groups spent significantly more time in the open arms than the Con group, suggesting that both AUS alone and the combination of AUS and BINT significantly altered time spent in open arms compared with Con conditions. Nonsignificant differences were observed between Con and BINT (*p* = 0.5163), BINT and AUS (*p* = 0.3173), BINT and AUS + BINT (*p* = 0.8395), and AUS and AUS + BINT (*p* = 0.9847) males. In female animals, analysis showed a significant difference between the Con and AUS groups (*p* = 0.0047) where the AUS females spent more time in the open arms, indicating that AUS alone significantly affected time spent in open arms in females. Nonsignificant differences were observed between Con and BINT (*p* = 0.1233), Con and AUS + BINT (*p* = 0.2933), BINT and AUS (*p* = 0.9157), BINT and AUS + BINT (*p* = 0.9997), and AUS and AUS + BINT (*p* = 0.6574) females. The assessment of sex differences revealed no significant differences between males and females in any of the treatment conditions: Con (*p* = 0.8973), AUS (*p* = 0.9708), BINT (*p* = 0.3679), and AUS + BINT (*p* > 0.9999). These results suggested that while there were overall sex differences in time spent in open arms, these differences were not pronounced within specific groups.

Assessment of the total time spent in the enclosed arms revealed AUS males and females spent less time in the enclosed arms than their Con counterparts (Fig. 5*D*). The three-way ANOVA revealed a significant main effect of sex (*p* < 0.0001, *F*_(1,88)_ = 16.94) and AUS (*p* = 0.0011, *F*_(1,88)_ = 11.33), but not BINT (*p* = 0.5918, *F*_(1,88)_ = 0.2897). There was a two-way interaction between AUS and BINT (*p* < 0.0001, *F*_(1,88)_ = 17.62), but not sex and AUS (*p* = 0.9693, *F*_(1,88)_ = 0.001486), or sex and BINT (*p* = 0.6406, *F*_(1,88)_ = 0.2194), nor was there a three-way interaction between sex, AUS, and BINT (*p* = 0.6279, *F*_(1,88)_ = 0.2366). Post hoc multiple comparisons revealed AUS males spent significantly less time in the enclosed arms compared with Con males (*p* = 0.0084). Nonsignificant differences were observed between the Con and BINT (*p* = 0.3287), Con and AUS + BINT (*p* = 0.7186), BINT and AUS (*p* = 0.7021), BINT and AUS + BINT (*p* = 0.9975), and AUS and AUS + BINT (*p* = 0.2950) groups. Similarly, AUS females spent significantly less time in the enclosed arms compared with Con females (*p* = 0.0175). Nonsignificant differences were observed between the Con and BINT (*p* = 0.2683), Con and AUS + BINT (*p* = 0.3529), BINT and AUS (*p* = 0.9316), BINT and AUS + BINT (*p* > 0.9999), and AUS and AUS + BINT (*p* = 0.8475) groups. There were no significant sex differences in any of the groups: Con (*p* = 0.5468), AUS (*p* = 0.7834), BINT (*p* = 0.3788), and AUS + BINT (*p* = 0.1394).

Assessment of total entries into the open arms revealed no differences between groups within each sex, but major sex differences within groups (Fig. 5*E*). The three-way ANOVA revealed a significant main effect of sex (*p* < 0.0001, *F*_(1,88)_ = 57.08), but no main effects of AUS (*p* = 0.2607, *F*_(1,88)_ = 1.282) or BINT (*p* = 0.6347, *F*_(1,88)_ = 0.2273). There were no two-way interactions between any of the variables: AUS and BINT (*p* = 0.0676, *F*_(1,88)_ = 3.424), sex and AUS (*p* = 0.7049, *F*_(1,88)_ = 0.1443), or sex and BINT (*p* = 0.1203, *F*_(1,88)_ = 2.460), nor was there a three-way interaction between sex, AUS, and BINT (*p* = 0.8648, *F*_(1,88)_ = 0.02918). Post hoc multiple comparisons showed no significant differences between any of the male groups: Con versus BINT (*p* = 0.6424), Con versus AUS (*p* = 0.825), Con versus AUS + BINT (*p* = 0.694), BINT versus AUS (*p* > 0.9999), BINT versus AUS + BINT (*p* > 0.9999), and AUS versus AUS + BINT (*p* > 0.9999). There were also no significant differences between groups in the female animals: Con versus BINT (*p* = 0.9998), Con versus AUS (*p* = 0.8691), Con versus AUS + BINT (*p* > 0.9999), BINT versus AUS (*p* = 0.9802), BINT versus AUS + BINT (*p* = 0.9974), and AUS versus AUS + BINT (*p* = 0.7313). These results suggested that in male and female animals, neither AUS nor BINT, alone or in combination, significantly affected the total number of entries into the open arms. While there were no differences between groups within each sex, there were significant sex differences with females experiencing more total entries into the open arms compared with their male counterparts: Con (*p* = 0.0012), AUS (*p* = 0.0008), and BINT (*p* = 0.0197). While the AUS + BINT females did have more entries into the open arms than their male counterparts, these differences were not statistically significant (*p* = 0.0837).

Assessment of average time per entry into the open arms revealed AUS and AUS + BINT males spent more time on average in the open arms than Con animals (Fig. 5*F*). The three-way ANOVA revealed significant main effects of sex (*p* = 0.0107, *F*_(1,88)_ = 6.796) and AUS (*p* < 0.0001, *F*_(1,88)_ = 20.23) but not BINT (*p* = 0.2904, *F*_(1,88)_ = 1.131). There were two-way interactions between AUS and BINT (*p* = 0.0009, *F*_(1,88)_ = 11.82) and sex and AUS (*p* = 0.036, *F*_(1,88)_ = 4.536), but not sex and BINT (*p* = 0.4304, *F*_(1,88)_ = 0.6276). Additionally, there was a three-way interaction between sex, AUS, and BINT (*p* = 0.0388, *F*_(1,88)_ = 4.402). Post hoc multiple comparisons further elucidated these results. The AUS (*p* < 0.0001) and AUS + BINT (*p* = 0.0311) males spent more time per entry into the open arms compared with Con males. This suggested that both AUS alone and the combination of AUS and BINT significantly altered the average time spent per entry compared with Con conditions. Interestingly AUS males spent more time per entry into the open arms compared with their BINT counterparts (*p* = 0.0391) but AUS + BINT males did not (*p* = 0.9991), suggesting experiencing BINT mitigated some of the effects of AUS on average time per entry. Nonsignificant differences were observed between Con and BINT (*p* = 0.1195) and AUS and AUS + BINT (*p* = 0.1537) males. In female animals, there were no significant differences between groups: Con and BINT (*p* = 0.7453), Con and AUS + BINT (*p* = 0.3868), Con and AUS (*p* = 0.6024), BINT and AUS (*p* > 0.9999), BINT and AUS + BINT (*p* = 0.9993), and AUS and AUS + BINT (*p* > 0.9999) females. When assessing sex differences, only AUS males and females were significantly different (*p* = 0.007) with males spending more time per entry than the females. There were no significant differences between the sexes in the other groups: Con (*p* > 0.9999), BINT (*p* = 0.9816), and AUS + BINT (0.9752). Together this suggests that, with respect to open-arm exploration, AUS affected males and females differently.

Assessment of time spent in the center of the EPM arena as a decision-making zone revealed that AUS + BINT had a major influence on the amount of time males spend in the center but no effect on females (Fig. 5*G*). The three-way ANOVA revealed main effects of sex (*p* = 0.0045, *F*_(1,88)_ = 8.522) and BINT (*p* = 0.008, *F*_(1,88)_ = 7.373), but not AUS (*p* = 0.7312, *F*_(1,88)_ = 0.1188). There was also a two-way interaction between sex and BINT (*p* = 0.0003, *F*_(1,88)_ = 14.38) but no interactions between AUS and BINT (*p* = 0.2049, *F*_(1,88)_ = 1.631) or sex and AUS (*p* = 0.1005, *F*_(1,88)_ = 2.756). Additionally, there was no three-way interaction between sex, AUS, and BINT (*p* = 0.9788, *F*_(1,88)_ = 0.0007129). Post hoc multiple comparisons revealed that AUS + BINT males spent less time in the center than Con (*p* = 0.0082) and AUS males (*p* = 0.0041). Nonsignificant differences were observed in males between the Con and BINT (*p* = 0.2218), Con and AUS (*p* > 0.9999), BINT and AUS (*p* = 0.1665), and BINT and AUS + BINT (*p* = 0.866) groups. These results suggested that in males, the combination of AUS and BINT significantly affected impulsivity, while individual factors had less pronounced effects. In females, there were no significant differences between any of the groups: Con versus BINT (*p* = 0.9384), Con versus AUS (*p* = 0.7456), Con versus AUS + BINT (*p* = 0.764), BINT versus AUS (*p* = 0.9997), BINT versus AUS + BINT (*p* > 0.9999), and AUS versus AUS + BINT (*p* > 0.9999). These results indicated that in female animals, neither AUS nor BINT, alone or in combination, significantly affected the total time spent in the center of the arena. The assessment of sex differences only revealed a significant difference between Con males and females (*p* = 0.0058) with males spending more time in the center than females. There were no other significant differences between AUS (*p* = 0.2072), BINT (*p* = 0.9999), or AUS + BINT (0.8691) males and females. These results suggested that while there are sex differences in baseline behavior, these differences were mitigated under AUS or BINT conditions.

### The combination of AUS and BINT in males and females led to decreased time spent in the center of the OF test arena

**Figure 6.**
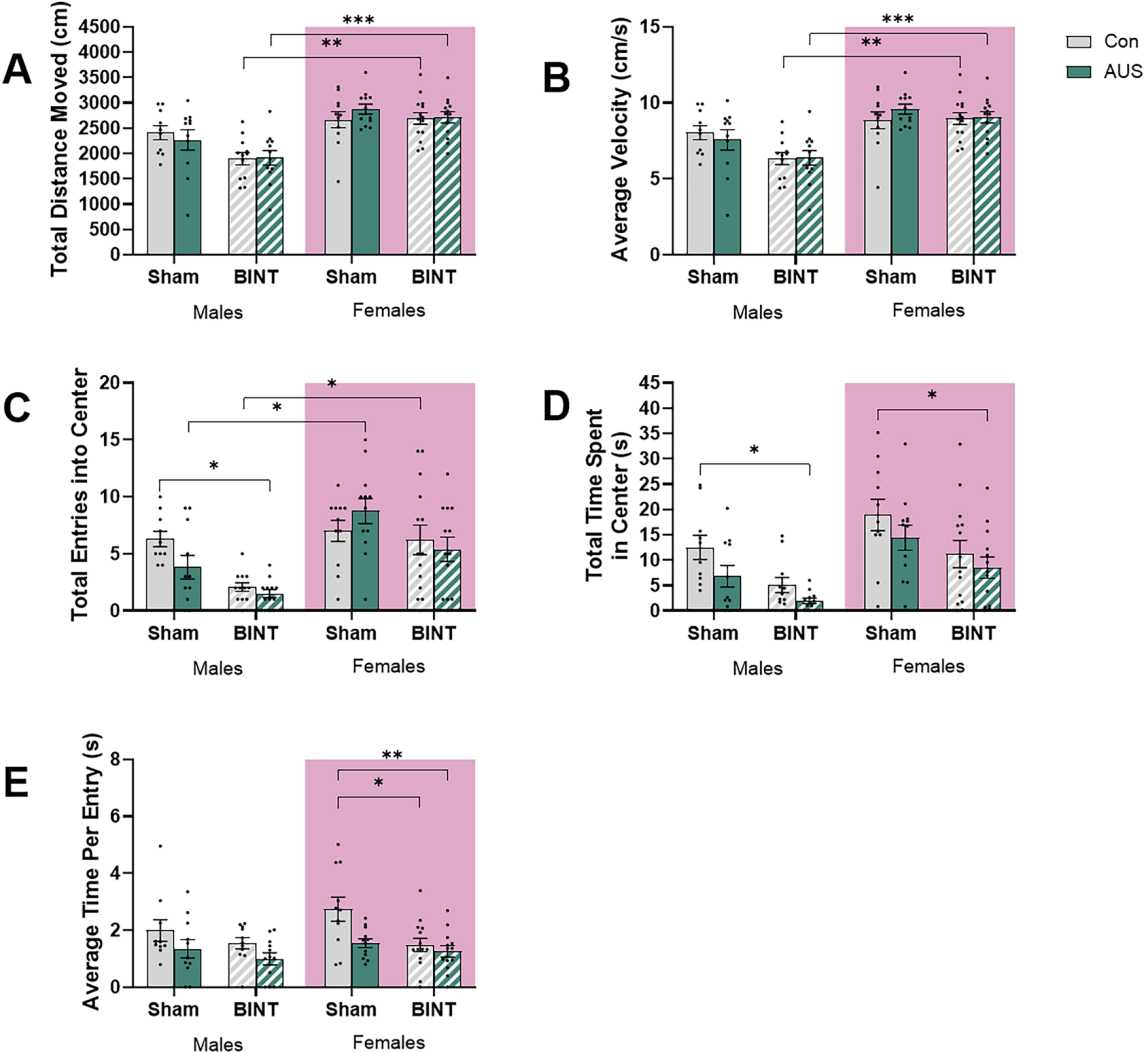
OF testing of male and female Sprague Dawley rats 30 d postinjury showed stress prior to a BINT reduced the total time spent in the center of the arena for both sexes despite the increased exploratory behavior of the females compared with the males. ***A***, Total distance moved measured in centimeters. ***B***, Average velocity measured in centimeters per second. ***C***, Total number of entries into the center of the arena. ***D***, Total time spent in the center of the arena in seconds. ***E***, Average time per entry calculated as seconds per entry (males, Con *n* = 10, AUS *n* = 11, BINT *n* = 12, AUS + BINT *n* = 12; females, Con *n* = 11, AUS *n* = 12, BINT *n* = 14, AUS + BINT *n* = 13).

Assessment of total distance moved revealed that there were no significant differences between experimental groups within each sex (Fig. 6*A*). A three-way ANOVA revealed main effects of BINT (*p* = 0.0111, *F*_(1,87)_ = 6.741) and sex (*p* < 0.0001, *F*_(1,87)_ = 40.26) but not AUS (*p* = 0.7795, *F*_(1,87)_ = 0.07884). There were no two-way interactions between AUS and BINT (*p* = 0.9426, *F*_(1,87)_ = 0.005219), sex and AUS (*p* = 0.3528, *F*_(1,87)_ = 0.8728), or sex and BINT (*p* = 0.0611, *F*_(1,87)_ = 3.600), nor was there a three-way interaction between sex, AUS, and BINT (*p* = 0.3781, *F*_(1,87)_ = 0.7847). Post hoc multiple comparisons showed no significant differences between any of the male groups: Con versus BINT (*p* = 0.1868), Con versus AUS (*p* = 0.997), Con versus AUS + BINT (*p* = 0.2186), BINT versus AUS (*p* = 0.5568), BINT versus AUS + BINT (*p* > 0.9999), and AUS versus AUS + BINT (*p* = 0.6099). Additionally, there were no significant differences between female groups: Con versus BINT (*p* > 0.9999), Con versus AUS (*p* = 0.9607), Con versus AUS + BINT (*p* > 0.9999), BINT versus AUS (*p* = 0.9728), BINT versus AUS + BINT (*p* > 0.9999), and AUS versus AUS + BINT (*p* = 0.9893). These results indicated that with male and female animals, neither AUS nor BINT, alone or in combination, significantly affected the total distance traveled. Assessment of sex differences revealed significant differences between BINT (*p* = 0.0011) and AUS + BINT (*p* = 0.0012) males and females with females traveling further. While there were no significant differences between Con (*p* = 0.9175) male and female animals, the AUS (*p* = 0.0514) females traveled further than the males, but the difference did not reach significance. These results suggested that while there are no significant sex differences in baseline behavior, the experimental conditions (AUS, BINT, or their combination) elicit different responses in the total distance traveled between males and females.

Assessment of average velocity revealed that there were no significant differences between experimental groups within each sex (Fig. 6*B*). A three-way ANOVA revealed main effects of BINT (*p* = 0.0126, *F*_(1,87)_ = 6.491) and sex (*p* < 0.0001, *F*_(1,87)_ = 39.17) but not AUS (*p* = 0.7615, *F*_(1,87)_ = 0.09268). There were no two-way interactions between AUS and BINT (*p* = 0.9205, *F*_(1,87)_ = 0.01002), sex and AUS (*p* = 0.3416, *F*_(1,87)_ = 0.9143), or sex and BINT (*p* = 0.0598, *F*_(1,87)_ = 3.638), nor is there a three-way interaction between sex, AUS, and BINT (*p* = 0.3683, *F*_(1,87)_ = 0.8180). Post hoc multiple comparisons showed no significant differences between any of the male groups: Con versus BINT (*p* = 0.1959), Con versus AUS (*p* = 0.9971), Con versus AUS + BINT (*p* = 0.2271), BINT versus AUS (*p* = 0.5691), BINT versus AUS + BINT (*p* > 0.9999), and AUS versus AUS + BINT (*p* = 0.6196). Additionally, there were no significant differences between any of the female groups: Con versus BINT (*p* > 0.9999), Con versus AUS (*p* = 0.951), Con versus AUS + BINT (*p* > 0.9999), BINT versus AUS (*p* = 0.9738), BINT versus AUS + BINT (*p* > 0.9999), and AUS versus AUS + BINT (*p* = 0.9897). These results indicated that with both male and female animals, neither AUS nor BINT, alone or in combination, significantly affected the average velocity. Assessment of sex differences revealed significant differences between BINT (*p* = 0.0013) and AUS + BINT (*p* = 0.0014) males and females with females traveling at a higher velocity. While there were no significant differences between Con (*p* = 0.9368) male and female animals, the AUS (*p* = 0.0553) females travel faster than the males but the difference did not reach significance. These results suggested that while there are no significant sex differences in baseline behavior, the experimental conditions (AUS, BINT, or their combination) elicit different responses in average velocity between males and females.

Assessment of total number of entries into the center of the arena revealed that AUS + BINT males spent less time in the center of the arena compared with Con animals (Fig. 6*C*). A three-way ANOVA revealed main effects of BINT (*p* = 0.0002, *F*_(1,87)_ = 15.65) and sex (*p* < 0.0001, *F*_(1,87)_ = 25.52) but not AUS (*p* = 0.4294, *F*_(1,87)_ = 0.6304). There were no two-way interactions between AUS and BINT (*p* = 0.8015, *F*_(1,87)_ = 0.06359), sex and AUS (*p* = 0.1437, *F*_(1,87)_ = 2.177), or sex and BINT (*p* = 0.3800, *F*_(1,87)_ = 0.7787), nor was there a three-way interaction between sex, AUS, and BINT (*p* = 0.1010, *F*_(1,87)_ = 2.748). Post hoc multiple comparisons showed a significant difference between Con and AUS + BINT males (*p* = 0.0204), suggesting that the combination of AUS and BINT significantly reduced the number of entries into the center compared with Con conditions. Nonsignificant differences were observed between male Con and BINT (*p* = 0.0649), Con and AUS (*p* = 0.6652), BINT and AUS (*p* = 0.9078), BINT and AUS + BINT (*p* = 0.9999), and AUS and AUS + BINT (*p* = 0.6901) groups. Alternatively, the females had no significant differences between groups: Con versus BINT (*p* = 0.9989), Con versus AUS (*p* = 0.9038), Con versus AUS + BINT (*p* = 0.9287), BINT versus AUS (*p* = 0.5092), BINT versus AUS + BINT (*p* = 0.9978), and AUS versus AUS + BINT (*p* = 0.1826). These results indicated that in female animals, neither AUS nor BINT, alone or in combination, significantly affected the number of entries into the center of the arena. Assessment of sex differences revealed significant differences between males and females in the AUS (*p* = 0.0116) and BINT (*p* = 0.038) groups where females had significantly more entries than males. No significant differences were observed in the Con (*p* = 0.9997) or AUS + BINT (*p* = 0.0727) groups. These results support that while there were no significant sex differences in baseline behavior or response to AUS + BINT, AUS and BINT females had more entries into the center than their male counterparts.

Assessment of total time spent in the center of the arena revealed that AUS + BINT males and females spent less time in the center compared with the Con animals (Fig. 6*D*). A three-way ANOVA revealed main effects of AUS (*p* = 0.0134, *F*_(1,88)_ = 6.377), BINT (*p* < 0.0001, *F*_(1,88)_ = 16.76), and sex (*p* < 0.0001, *F*_(1,88)_ = 17.85). There were no two-way interactions between AUS and BINT (*p* = 0.4944, *F*_(1,88)_ = 0.4708), sex and AUS (*p* = 0.7912, *F*_(1,88)_ = 0.07053), or sex and BINT (*p* = 0.8318, *F*_(1,88)_ = 0.04537), nor was there a three-way interaction between sex, AUS, and BINT (*p* = 0.9011, *F*_(1,88)_ = 0.01554). Post hoc multiple comparisons showed a significant difference between Con and AUS + BINT males (*p* = 0.0388), indicating that the combination of AUS and BINT significantly reduced time spent in the center compared with Con conditions. Nonsignificant differences were observed between Con and BINT (*p* = 0.3115), Con and AUS (*p* = 0.6926), BINT and AUS (*p* = 0.9993), BINT and AUS + BINT (*p* = 0.971), and AUS and AUS + BINT (*p* = 0.8005) males. Additionally, there were significant differences between Con and AUS + BINT females (*p* = 0.03) but not between the Con and BINT (*p* = 0.218), Con and AUS (*p* = 0.8608), BINT and AUS (*p* = 0.9618), BINT and AUS + BINT (*p* = 0.9849), and AUS and AUS + BINT (*p* = 0.5413) groups. Together these results indicated that in both male and female animals, the combination of AUS + BINT had a more pronounced effect on time spent in the center than AUS or BINT alone. There were no significant differences between males and females in any of the groups: Con (*p* = 0.5541), AUS (*p* = 0.2674), BINT (*p* = 0.4472), or AUS + BINT (*p* = 0.4052).

Analysis of the average time spent in the center of the arena per entry revealed that BINT and AUS + BINT females spent less time per entry in the center of the arena compared with their Con counterparts (Fig. 6*E*). A three-way ANOVA revealed main effects of AUS (*p* = 0.0013, *F*_(1,88)_ = 11.10) and BINT (*p* = 0019, *F*_(1,88)_ = 10.22), but not sex (*p* = 0.1102, *F*_(1,88)_ = 2.603). There were no two-way interactions between AUS and BINT (*p* = 0.1359, *F*_(1,88)_ = 2.266), sex and AUS (*p* = 0.6759, *F*_(1,88)_ = 0.1760), or sex and BINT (*p* = 0.3902, *F*_(1,88)_ = 0.7456), nor was there a three-way interaction between sex, AUS, and BINT (*p* = 0.2996, *F*_(1,88)_ = 1.089). Post hoc multiple comparisons showed that there were no significant differences between groups in the males: Con versus BINT (*p* = 0.8709), Con versus AUS (*p* = 0.7583), Con versus AUS + BINT (*p* = 0.2034), BINT and AUS (*p* > 0.9999), BINT versus AUS + BINT (*p* = 0.9123), and AUS versus AUS + BINT (*p* = 0.9833). In the female animals, both the BINT (*p* = 0.024) and AUS + BINT (*p* = 0.00043) females spent less time per entry than the Con females. While the AUS females spent less time per entry than the Con females, the difference did not reach significance (*p* = 0.0511). Additionally, there were no significant differences between AUS and BINT (*p* > 0.9999), AUS and AUS + BINT (*p* = 0.994), or BINT and AUS + BINT (*p* = 0.9982) females. There were no significant sex differences within groups: Con (*p* = 0.588), AUS (*p* = 0.9996), BINT (*p* > 0.9999), and AUS + BINT (*p* = 0.9967). These results further support the lack of an overall main effect of sex difference observed in the three-way ANOVA.

## Discussion

Our study's findings revealed important insights into the effects of BINT and AUS on animal behavior, with a particular focus on mobility and exploratory patterns. Crucially, we observed no significant motor deficits attributable to either BINT or AUS across all behavioral tests, indicating that the treatments did not impair basic locomotor function. This observation is fundamental to the interpretation of our results, as it allows us to attribute behavioral changes to cognitive or emotional factors rather than physical limitations. While we found no major differences in total distance traveled or average velocity between groups within each sex, our data revealed intriguing sex-specific responses across different behavioral paradigms. These sex differences in mobility and exploratory behavior warrant careful consideration and may provide valuable insights into the differential effects of BINT and AUS on male and female subjects.

In the EPM test, we observed that BINT females generally exhibited greater mobility than their male counterparts, covering longer distances at higher velocities. Interestingly, this sex difference was not apparent in the Con, AUS, and AUS + BINT groups, suggesting a potential interaction between AUS treatment and sex-specific behavioral responses. The mitigation of sex differences in these groups appears to stem from slight increases in male mobility and decreases in female mobility compared with their respective controls. Contrastingly, in the 3-CS, 3-CSN, and OF, we observed that the AUS, BINT, and AUS + BINT females demonstrated increased mobility compared with their male counterparts, despite no observable sex differences in the control group. This heightened mobility in BINT females could potentially be indicative of increased anxiety-like behavior. It is also noteworthy that BINT males exhibited reduced mobility during the 3-CS, which we attribute to an increase in time spent investigating the stranger's chamber rather than a decrease in overall exploratory drive. These complex findings underscore the importance of considering both sex and specific behavioral contexts when evaluating the effects of BINT and AUS. This discussion will delve deeper into exploring potential mechanisms underlying these sex-specific responses and their implications for our understanding of BINT and AUS and their interactions.

Anhedonia is commonly defined as the inability to experience pleasure or enjoyment from activities that would normally be pleasurable and is a core symptom of depression (Serretti, 2023). Social anhedonia (SA) is associated with a variety of social and emotional difficulties including less social skill, contact, interest, and pleasure as well as less social coping strategies (Dodell-Feder and Germine, 2018). For rats, social interactions are typically considered rewarding (Contestabile et al., 2021); however, the impairment of social functioning is often seen as a sign of SA and depression-like behaviors (Hirschfeld et al., 2000). By using the 3-CS test, we were able to test for SA by assessing both sociability as well as preference for social novelty. In this context, “sociability” was defined as the inclination to spend time with another rodent, as opposed to spending time alone while “preference for social novelty” was defined as a predisposition to spend time with a previously unencountered rodent rather than a familiar one (Kaidanovich-Beilin et al., 2011). The comparison between the time spent on the two sides determines whether a group of animals displays sociability or not while the time spent sniffing the novel animal provides a corroborative and more specific measure of social investigation (Yang et al., 2016). During this study, male and female Sprague Dawley rats were tested for sociability and preference for social novelty at 28 d post-BINT.

Our study revealed intriguing differences in sociability and social novelty behaviors across groups and between sexes, highlighting the complex interplay between BINT and AUS in social interactions. BINT had the most pronounced effects on sociability, but interestingly, these effects were sex-dependent. Male BINT subjects exhibited increased sociability, contrasting with the social withdrawal typically observed in TBI cases (May et al., 2017; Collins et al., 2020). This unexpected finding warrants further investigation to understand the underlying mechanisms. Conversely, female BINT subjects displayed decreased sociability, aligning more closely with established literature. This stark contrast between male and female responses to BINT underscores the importance of considering sex as a biological variable in TBI research.

While all AUS-treated groups showed reduced social interaction with the stranger animal, only male AUS + BINT animals exhibited significantly decreased sociability compared with their Con cohorts. This observation aligns with previous findings that both psychological stress and TBI separately can lead to decreased social behavior (May et al., 2017; von Dawans et al., 2018, 2021; Collins et al., 2020) and may also indicate an additive effect not seen in AUS or BINT alone. The lack of significant effects in other AUS groups might be attributed to the timing of testing, suggesting a potential recovery of social deficits by 28 d postinjury.

The effects of BINT on social novelty preferences were equally striking and sex-dependent. Male BINT subjects showed a preference for familiar animals over novel ones, while female BINT subjects exhibited the opposite tendency. This sex difference, while substantial, is not entirely unexpected given the known differential effects of TBI on males and females. The medial amygdala (MeA) plays a crucial role in animal recognition and social interactions (Bergan et al., 2014). TBI and traumatic stress often disrupt amygdalar function (Nordman et al., 2020; Gimbel et al., 2023), which could contribute to the observed behavioral changes. The differential activation of MeA neurons in male and female mice exposed to predator urine (Kikusui et al., 2018) might offer insights into the sex-specific social behaviors observed following traumatic stressors. This could also contribute to the near loss of preference for novel versus familiar animals in AUS + BINT females which could be driven by either impaired animal–animal recognition or short-term memory formation (Girgis et al., 2016). Further investigation is needed to determine whether this lack of preference stems from diminished novelty-seeking behavior or memory impairment. However, the AUS + BINT groups showed marked differences from the BINT-only groups in both sexes, indicating that the molecular mechanisms of stress may have a dominant effect over TBI in terms of sociability and social novelty preferences, particularly if the stress comes premorbid to an injury. Interestingly, AUS alone did not significantly affect social novelty behavior at 27–28 d postinjury, suggesting a potential recovery of AUS-induced social deficits by this timepoint. In conclusion, our findings highlight the complex and sex-dependent effects of BINT and AUS on sociability and social novelty preferences. These results emphasize the need for further research to elucidate the underlying mechanisms and potential interventions to address social deficits following TBI and acute stress.

It is important to note that behavioral readouts in the 3-CS task can be influenced by factors, such as anxiety-like behaviors (Lukas and de Jong, 2017). Anxious animals will either show a decrease in social preference when they are too anxious to explore their surroundings or they increase their social approach in an attempt to socially buffer their anxiety (Contestabile et al., 2021). To assess anxiety-like behavior in our animals, we used a combination of EPM and OF. Both EPM and OF are routinely used to evaluate emotional behavior in rodents by measuring general exploratory activity and avoidance of the aversive open arms of the maze (Schrader et al., 2018) and avoidance of the open center of the OF arena (Kraeuter et al., 2019). Relative distributions of time and behavioral performance in the open versus enclosed arms are used to interpret open-arm avoidance, which translates to clinically relevant negative emotional states like aversion, hypervigilance, risk tolerance, and anxiety (Schrader et al., 2018). Typically, rodents show avoidance of the open arms and a preference for the more protected enclosed arms comparable to the rodents’ preference for the outer-perimeter during the OF testing. However, our study revealed complex patterns of anxiety-like behaviors across treatment groups and between sexes, highlighting the intricate nature of assessing anxiety in rodent models of BINT and AUS.

During EPM testing, AUS and AUS + BINT males exhibited increased time in open arms, which could be interpreted as reduced anxiety. However, this behavior may instead reflect increased hypervigilance, risk tolerance, or impulsivity. The association between impulsivity and increased open-arm exploration (Pawluk and Koerner, 2013; Rico et al., 2016) supports this interpretation. Furthermore, male AUS + BINT animals spent less time in the maze center, a behavior linked to decision-making and increased impulsivity (Rico et al., 2016). Thus, the increased open-arm time in these groups may indicate heightened impulsivity rather than reduced anxiety, as impulsivity has been associated with anxiety-like behavior (Pawluk and Koerner, 2013). Notably, we observed consistent sex differences in EPM performance, with females showing more open-arm entries than males across all groups. This difference likely stems from the increased mobility of females, as evidenced by greater distance traveled, higher average velocity, and decreased average time per open-arm entry suggesting that this increased exploratory behavior may be driven by anxiety (Contestabile et al., 2021). This pattern of heightened female exploration was also observed in OF.

In the OF, traditionally interpreted as showing reduced anxiety when more time is spent in the center, we only observed the expected pattern of decreased center time and entries in AUS + BINT males and females. This limited effect could be due to the timing of the OF at 30 d postinjury. While some studies suggest that anxiety-like behaviors were observed ∼ 2 weeks to 1 month post-TBI in rodents (Tucker and McCabe, 2021), others propose that a more comprehensive assessment of behavioral changes, including anxiety-like behaviors, may require testing at 3–4 months post-TBI (Lassarén et al., 2023). This could further support the idea that by combining AUS and BINT, we are discovering an additive effect of combining psychological stress and TBI that would not normally be seen with these treatments alone. In conclusion, our findings underscore the complexity of assessing anxiety-like behaviors in rodent models of TBI and AUS. The observed sex differences and varied responses to treatments highlight the need for careful interpretation of behavioral data and the importance of using multiple tests to comprehensively evaluate anxiety-like states. Future studies examining both acute and chronic timepoints may provide further insights into the development and persistence of anxiety-like behaviors following TBI and AUS.

It is important to note distinct caveats to our behavioral testing. Behavior testing on rodents was conducted under standard lighting, which could have influenced anxiety-like behaviors due to their preference for dark spaces. The EPM and OF tests, designed to assess anxiety-like behavior, measure different aspects of anxiety and exploratory behavior. The EPM test is based on rodents’ natural aversion to open and elevated spaces, while the OF test assesses thigmotaxis in an open, enclosed arena (Shoji and Miyakawa, 2021). Exposure to a novel environment, such as during EPM testing, could increase motor activity and open-arm exploration (Shoji and Miyakawa, 2021). However, OF testing does not occur in a novel environment. AUS males and females, as well as AUS + BINT males, showed decreased anxiety-like behaviors during EPM by spending more time exploring the open arms compared with their Con counterparts. This supports the concept of increased risk-taking behavior during EPM. Despite the room lighting, these groups showed increases in anxiety-like behavior during OF testing. Additionally, the interpretation of OF and EPM results in the context of anxiety is complex. During OF, common anxiolytic drugs do not consistently increase center time (Thompson et al., 2015), and the test has been criticized for its inability to differentiate between locomotion, exploration, and anxiety (Cheeta et al., 2001). Therefore, conclusions about anxiety states from OF should be corroborated with more specific anxiety tests like the light/dark box or EPM (Tucker and McCabe, 2021). It is also common for OF and EPM to have contradictory results with one test showing anxiety-like behavior while the other does not; this reinforces the need for multiple behavior tests to assess anxiety.

Collectively, our findings highlight the importance of considering sex as a biological variable in neurotrauma and stress research. It was demonstrated that males and females can respond differently to the same stressors or injuries, potentially through different neurobiological mechanisms. This has important implications for understanding and treating AUS-related disorders and neurotrauma in clinical populations, where sex-specific approaches may be necessary. Taken together, the differences in behavioral outcomes between males and females across all behavioral tests suggest that unlike the males, the anxiety-like behavior in the females may not be presenting itself in the form of risk-taking or impulsivity-related behaviors but in the form of social anxiety. Further behavioral assessment of social interaction and memory is needed to fully appreciate the influence of prestress on TBI outcomes in females.

It has been shown that patients who have a history of anxiety or depression prior to a TBI are more likely to develop worsening or secondary psychiatric disorders (Gould et al., 2011; Barker-Collo et al., 2013; Scholten et al., 2016). Despite this and the increasing prevalence of anxiety- and mood-related disorders, very few studies have attempted to understand the effects that these changes would have on a subsequent TBI. The importance of evolving TBI research to include preexisting conditions such as stress, anxiety, and depression is vital to develop clinically relevant preclinical TBI models. It is evident that further studies are necessary to fully understand the effects of acute and chronic stress on the outcomes of neurotrauma, not only for patients who have high-stress occupations such as military personnel, first responders, and doctors but also for all patients.

## Synthesis

Reviewing Editor: Viji Santhakumar, University of California Riverside Decisions are customarily a result of the Reviewing Editor and the peer reviewers coming together and discussing their recommendations until a consensus is reached. When revisions are invited, a fact-based synthesis statement explaining their decision and outlining what is needed to prepare a revision will be listed below. The following reviewer(s) agreed to reveal their identity: Idrish Ali.

The study combines acute stress followed immediately by repeated mild blast TBI to address an important question of relevance to military TBI. The study specifically examines social behavior, anxiety and risk-taking behavior after the insults delivered individually and in combination. The study demonstrates that a stress prior to a blast TBI can affect the outcome of the TBI. The authors have performed considerable revisions to address prior concerns. However, some lingering concerns about statistical analysis remain. Additionally, the claim that the study relevance extends beyond the military is weak given the extensive literature enumerating differences between blast TBI and civilian impact TBI.

1. While the reviewers agree that stress is part of everyday life and can impact the outcomes of TBI, it is a stretch to extend results of current study to the civilian population in general. That is because it is established that blast TBI and civilian (impact-based) TBI share very few neuropathological features (extensively documented by Brody and colleagues). Thus, it may be best to limit generalizing to civilian injuries.

2. The presentation of data is not systematic the authors present main effects in some cases and ignore them in others and post hoc effects in some not others. this needs to be systematically addressed. It is clear in all the graphs of figure 3C-H that there is a main effect of AUS or stress that leads to lower social interest with the stranger animal chamber (also expected from literature reports) and that there is a statistical interaction with the Bint group that somehow increased the social interest in these animals. This is also evident by the order of writing in the result text for example in the section 'BINT males were more socially motivated than AUS and AUS+BINT animals during 3-ChS', where the authors presented the post hoc interactions first without any reference to the main two way Anova outcomes which are only reported later in the text perhaps an afterthought? In some cases, the authors even ignore the significant main outcomes if the post hoc comparisons were not significant. Example of this is on page 10, line 213. Two-way Anova showed that AUS led to significant effect (reduction) in the social interaction. It is not reported on the graphs, not possible without a group analysis graph.

3. The same data is presented twice making the text tedious. Individual figures to analyze differences in males and females is redundant when the combined analysis is provided. The authors should consider merging the presentation to avoid duplicating data sets. Data in figures 3 and 4 are in figure 5, data in figures 6 and 7 are in figure 8, and so on. One figure is sufficient for each outcome. Using same data more than once for different statistical analyses is not appropriate.

4. Although the authors have performed ANOVA for data analysis, as suggested, the graphs are still presented in the column format and not the 2 by 2 grouped format as provided by Prism. The column format of the output presented in the manuscript makes it confusing to interpret the Two-way ANOVA results. The authors should consider two factor matrix, which would be Bint vs No Bint and AUS vs No AUS to present the graphs appropriately. The authors do not report a post-hoc analysis following the 2- or 3-way ANOVA (the authors conduct a main effects ANOVA). The authors should consider two factor matrix, which would be Bint vs No Bint and AUS vs No AUS to present the graphs appropriately. Figures suggest that pairwise comparisons were conducted. However, these data are not presented.

5. Why was a two-way ANOVA not conducted for the CORT analysis?

6. For the social behavior assay (3 chamber test) why are data from chamber times being presented when the discrimination index covers the parameters?

7. The discussion needs significant revision as it reads as a rehash of the results section. Without going overboard on the speculation, the authors should include a section on what is it about the stress that could influence the TBI related outcomes. This is particularly important because the effect of pre-injury stress is not the same across all injury-related outcomes.

8. The color scheme i.e., black and red used in the figure 1 is a bit confusing. It is not following a pattern for the AUS/BINT; whether a black or a red color represent if BINT was delivered or not delivered?
